# *Nats’eji* (*healing*): Examining patient and provider experiences with hospital-based Indigenous wellness services in Northwest Territories, Canada

**DOI:** 10.17269/s41997-024-00959-6

**Published:** 2024-11-11

**Authors:** Sophie Isabelle Grace Roher, Paul Andrew, Susan Chatwood, Kimberly Fairman, Tracey Galloway, Angela Mashford-Pringle, Jennifer L. Gibson

**Affiliations:** 1 Department of Anthropology, University of Toronto Mississauga, Mississauga, ON Canada; 2 Institute for Circumpolar Health Research, Yellowknife, Northwest Territories, Canada; 3 School of Public Health, University of Alberta, Edmonton, AB Canada; 4 School of Public Health and Social Policy, University of Victoria, Victoria, BC Canada; 5 Dalla Lana School of Public Health, University of Toronto, Toronto, ON Canada; 6 Joint Centre for Bioethics, University of Toronto, Toronto, ON Canada

**Keywords:** Indigenous population, Indigenous medicine, Traditional medicine, Self-determination, Holistic health, Hospital care, Quality of care, Population indigène, Médecine indigène, Médecine traditionnelle, Autodétermination, Santé holistique, Soins hospitaliers, Qualité des soins de santé

## Abstract

**Objective:**

This study aimed to examine how Indigenous patients and biomedical healthcare providers understand and experience the Indigenous wellness services at a hospital in the Northwest Territories.

**Methods:**

The qualitative study (May 2018–June 2022) was overseen by a regional Indigenous Community Advisory Committee. Guided by Two-Eyed Seeing and post-colonial theory, the study employed a community-engaged research design, and included two strategies for data generation: (1) interviews with Indigenous Elders, patient advocates, biomedical healthcare providers, policy makers, and hospital administrators (*n* = 41), and (2) iterative sharing circles with Indigenous Elders (*n* = 4). Data from the interviews and first sharing circle were transcribed, thematically analyzed, and presented to the sharing circle Elders for validation.

**Results:**

The study revealed three overarching and related themes: (1) Elders and patient advocates emphasized that while the Indigenous wellness services at the hospital play a pivotal role connecting patients with cultural supports, the hospital was still not effectively bringing Indigenous healing practices into hospital care; (2) participants identified that structural factors (i.e., policy and governance decisions) shaped patients’ experiences with the wellness services; and (3) participants underscored that deeply rooted forces (i.e., racism, colonialism, and biomedical dominance) inhibit the integration of Indigenous healing practices.

**Conclusion:**

The findings extend understandings of hospital-based Indigenous wellness services by surfacing relationships between deeply rooted forces, organizational structures, and Indigenous patients’ experiences. Altogether, they suggest that to advance care for Indigenous patients and improve the integration of Indigenous healing practices, a system-wide transformation is necessary, which includes Indigenous governance at the hospital and a recognition of the value of Indigenous healing practices.

## Résumé

**Objectif:**

Déterminer comment les patients autochtones et les fournisseurs de soins de santé biomédicaux interprètent et vivent les services de bien-être autochtone dans un hôpital des Territoires du Nord-Ouest.

**Méthode:**

Guidée par la « vision à deux yeux » et par la théorie postcoloniale, une étude qualitative (mai 2018–juin 2022) menée sous la supervision d’un comité consultatif communautaire autochtone régional a employé un plan de recherche faisant appel aux communautés et incluant deux stratégies de production de données : 1) des entretiens avec des aînés autochtones, des défenseurs des patients, des fournisseurs de soins de santé biomédicaux, des responsables des politiques et des administrateurs de l’hôpital (n = 41); et 2) des cercles de partage itératifs avec des aînés autochtones (n = 4). Les données des entretiens et du premier cercle de partage ont été transcrites, analysées par thème, puis présentées aux aînés des cercles de partage, qui les ont validées.

**Résultats:**

L’étude a mis au jour trois grands thèmes connexes : 1) selon les aînés et les défenseurs des patients, les services de bien-être autochtone de l’hôpital jouent un rôle central en mettant les patients en contact avec des soutiens culturels, mais l’établissement n’intègre pas encore bien les pratiques de guérison autochtones dans les soins hospitaliers; 2) selon les participants, des facteurs structurels (les décisions de principe et de gouvernance) structurent l’expérience des services de bien-être vécue par les patients; et 3) les participants ont souligné que des forces profondes (racisme, colonialisme, domination du modèle biomédical) freinent l’intégration des pratiques de guérison autochtones.

**Conclusion:**

Les constatations de l’étude améliorent la compréhension des services de bien-être autochtone à l’hôpital en amenant à la surface les relations entre les forces profondes, les structures organisationnelles et l’expérience des patients autochtones. Ensemble, ces constatations indiquent que pour améliorer les soins aux patients autochtones et l’intégration des pratiques de guérison autochtones, il faut opérer une transformation de tout le système, ce qui comprend la gouvernance autochtone à l’hôpital et la reconnaissance de la valeur des pratiques de guérison autochtones.

## Introduction

### Study background

Research studies and reports have demonstrated the harmful and widespread impacts of Indigenous-specific racism in the Canadian healthcare system (Allan & Smylie, 2015; Cooper et al., 2021; Goodman et al., 2017; The Brian Sinclair Working Group, 2017). Scholars have repeatedly illustrated that when First Nations, Inuit, and Métis peoples (i.e., Indigenous peoples in what is now known as Canada) have negative experiences with the healthcare system, it can have detrimental impacts on health outcomes. Indigenous peoples who experience dismissal, discrimination, and racism in the healthcare system tend to anticipate difficult encounters with healthcare professionals; strategize about how to manage racism before entering healthcare environments; disengage, delay, or avoid seeking healthcare services; and underreport their symptoms to doctors and nurses (Allan & Smylie, 2015; Cooper et al., 2021; Goodman et al., 2017).

To improve Indigenous peoples’ healthcare experiences and reduce health inequities, national and international reports including the Truth and Reconciliation Commission (TRC) and the United Nations Declaration on the Rights of Indigenous Peoples (UNDRIP) have called for greater recognition of the value of Indigenous healing practices within the publicly funded healthcare system, and for improved collaboration between Indigenous and biomedical healthcare practitioners (Truth & Reconciliation Commission of Canada, 2015; United Nations General Assembly, 2007). These reports underscore that to achieve optimal health outcomes for Indigenous peoples, Indigenous knowledges and models of healing must be an integral part of contemporary healthcare options for Indigenous peoples and communities.

Hospitals in Canada have increasingly started to offer Indigenous wellness services, such as translation and interpretation, traditional foods, patient navigation, and access to cultural supports and medicines (Drost, 2019; Maar & Shawande, 2010; Manitowabi & Shawande, 2013; Walker et al., 2010). The limited but growing scholarship on hospital-based Indigenous wellness services has demonstrated strengths and challenges to integration of care. For example, Indigenous governance has been highlighted as a best practice in the literature. In their evaluations of the traditional medicines program at Noojmowin Teg Health Centre on Manitoulin Island, Maar & Shawande (2010) and Manitowabi and Shawande (2013) underscored the importance of having a traditional advisory working group of Elders to determine how Indigenous healing practices would be brought into clinical settings and to provide ongoing guidance and direction on the development of Indigenous healing protocols. Maar & Shawande (2010) asserted that the traditional advisory group was particularly critical in helping to address contentious issues, such as the potential regulation of Traditional Knowledge. Nevertheless, researchers also warn that when Indigenous knowledges and healing practices are brought into mainstream healthcare settings, Indigenous healing is at greater risk of being misused, tokenized, and/or controlled by non-Indigenous peoples and policies (Drost, 2019; Hill, 2003; Manitowabi & Shawande, 2013). To protect the integrity of Indigenous healing practices and knowledges, some Indigenous scholars have suggested that Elders and/or Knowledge Holders need to be the ones to define “the how, when, where, who, what and why of its utilization in the best service of Aboriginal peoples” (National Aboriginal Health Organization (NAHO), 2008, p. 17). That is, Indigenous peoples and communities need to be able to determine and define *whether* integration takes place, *what* integration looks like, and *how* it is managed and governed.

In this article, we use the term “biomedical” to refer to forms of healing derived from the biomedical model of health, which is commonly understood to have two essential features. First, the biomedical model understands illness and its causes to derive from biological, chemical, and physical attributes and thus often focuses on interventions that promote physiological health and/or prevent or treat physical diseases. Second, it emphasizes the scientific basis and “validity” of healing practices using mechanical, objective, and measurable methods (e.g., laboratory research) and thus prioritizes forms of knowledge and knowledge gathering that align with objectivism, positivism, and empiricism (Horrill et al., 2018; McEwen & Willis, 2014).

In 2015, authors SR and SC collaborated with the former Elders’ Advisory Council at the Stanton Territorial Health Authority on a scoping review of hospital-based Indigenous wellness models in Canada and Alaska to inform the creation of an Indigenous wellness centre beside Stanton Territorial Hospital (STH) in Yellowknife, Northwest Territories (NT). STH is the largest hospital in the NT. It offers in-person acute and ambulatory care to residents of the NT, where over 50% of the population are First Nations, Métis, or Inuit, and to residents of Western Nunavut, where over 85% of residents are Inuit (Government of the Northwest Territories, 2018; Statistics Canada, 2016). During our work together, the Elders underscored the need to better understand how Indigenous patients and biomedical healthcare providers understand and experience Indigenous wellness services in NT hospitals.

### Research objectives

Building on the Elders’ questions, our study sought to (1) describe participants’ understandings of and experiences with the Indigenous wellness services at STH; and (2) examine the factors that shape participants’ understandings and experiences with these services. Our hope was that an in-depth examination of patient and provider experiences would shed light on opportunities for and challenges to hospital-based Indigenous wellness services with the goal of advancing care for Indigenous patients at STH.

## Methods

### Study context

STH has an Indigenous Wellness Program, which is made up of eight full-time employees, including five Indigenous Patient Liaisons, a Traditional Foods Coordinator, and a Resident Elder. The Indigenous Wellness Program staff work on-site Monday–Friday from 8:30 am to 4:30 pm. They offer interpretation in seven of the NT’s nine official Indigenous languages; assist with patient navigation; provide patients with cultural supports such as crafts and sewing; and make a meal of traditional foods once a week and a snack of traditional foods once a week. Traditional foods are foods that are harvested and prepared according to local Indigenous knowledges and protocols. The Resident Elder visits patients at the hospital and holds talking circles and smudging ceremonies (i.e., ceremonies where sacred herbs are burned to cleanse a person physically, mentally, spiritually, and emotionally). Additionally, STH has implemented other changes to support the delivery of culturally safe care for Indigenous patients. For instance, the hospital has a non-denominational spiritual room on the first floor, which is surrounded by windows and equipped with a ventilation system so that individuals can smudge in the room.

Historically, STH also had an Elders’ Advisory Council. In 2007, the Minister of Health and Social Services at the territorial government created the Elders’ Advisory Council, which was made up of nine Elders and Knowledge Holders who represented the diversity of Indigenous cultures and languages in the NT. The Elders’ Advisory Council worked with Stanton Territorial Health Authority to create the Indigenous Wellness Program at STH in 2008. However, in 2016, the Elders’ Advisory Council was disbanded. The Elders’ Advisory Council has not yet been replaced at the hospital and represents a gap in Indigenous governance at STH.

Separate from the Indigenous Wellness Program at STH is a not-for-profit community organization in Yellowknife called the Arctic Indigenous Wellness Foundation (AIWF), which provides Indigenous healing practices at an on-the-land wellness camp. The AIWF is located about a 15-min drive from STH and informal relationships have started to develop between the AIWF and STH staff. Though the AIWF is not directly connected to the hospital, it is important to this research because the AIWF is exploring the potential of developing an Indigenous wellness centre beside STH.

To respond to the Elders’ objectives, SR worked with Elder Advisors to bring together an interdisciplinary team of Indigenous and non-Indigenous researchers. Two of the team members had worked with the former Elders’ Advisory Council. Altogether, the team brought perspectives in anthropology, nursing, health policy, and health ethics and expertise in northern and Indigenous health research, qualitative and community-based methods, and health services and policy research.

### Research ethics

The study (May 2018–June 2022) received research ethics approval from the University of Toronto, a northern research licence from Aurora Research Institute, and a research agreement with the Northwest Territories Health and Social Services Authority. The study was overseen throughout by an Indigenous Community Advisory Committee (CAC). The CAC was made up of two Dene Elders and an Inuk health systems researcher. Before applying for research ethics and starting data collection, SR worked with the CAC to draft and sign a Community Collaboration and Partnership Letter, which outlined the goals for the project, roles and responsibilities, and the ways that the study would respect the principles of reciprocity, relevance, responsibility, and respect.

### Research design and methods

Two-Eyed Seeing (TES) and post-colonial theory provided the theoretical grounding for the study. TES (*Etuaptmumk*) is a Mi’kmaw guiding principle, which recognizes that there are many ways of looking at the world; through the process of weaving between the strengths of Indigenous ways of knowing and non-Indigenous ways of knowing, one can develop a more nuanced and richer understanding (Bartlett et al., 2012). In this study, TES prompted us to see knowledge as co-constructed through conversation and storytelling*.* As such, we drew upon narrative research methods (i.e. sharing circles and interviews), which purposefully open space for Indigenous and non-Indigenous knowledge exchange (Kovach, 2010; Martin, 2009).

Additionally, post-colonial theory was chosen as a theoretical approach because it provides a language and framework to understand the relationship between knowledge and colonial power (Browne et al., 2005; Loomba, 2007). In this project, post-colonial theory prompted us to employ community-engaged research methods and work under the oversight of an Indigenous Community Advisory Committee (CAC) throughout. The CAC advised on all aspects of the research including the research questions, design, methods, and knowledge translation activities, and they helped ensure that the project adhered to the principles of Ownership, Control, Access, and Possession (OCAP), which are fundamental to Indigenous research in Canada (First Nations Information Governance Committee, 2024).

**Fig. 1:**
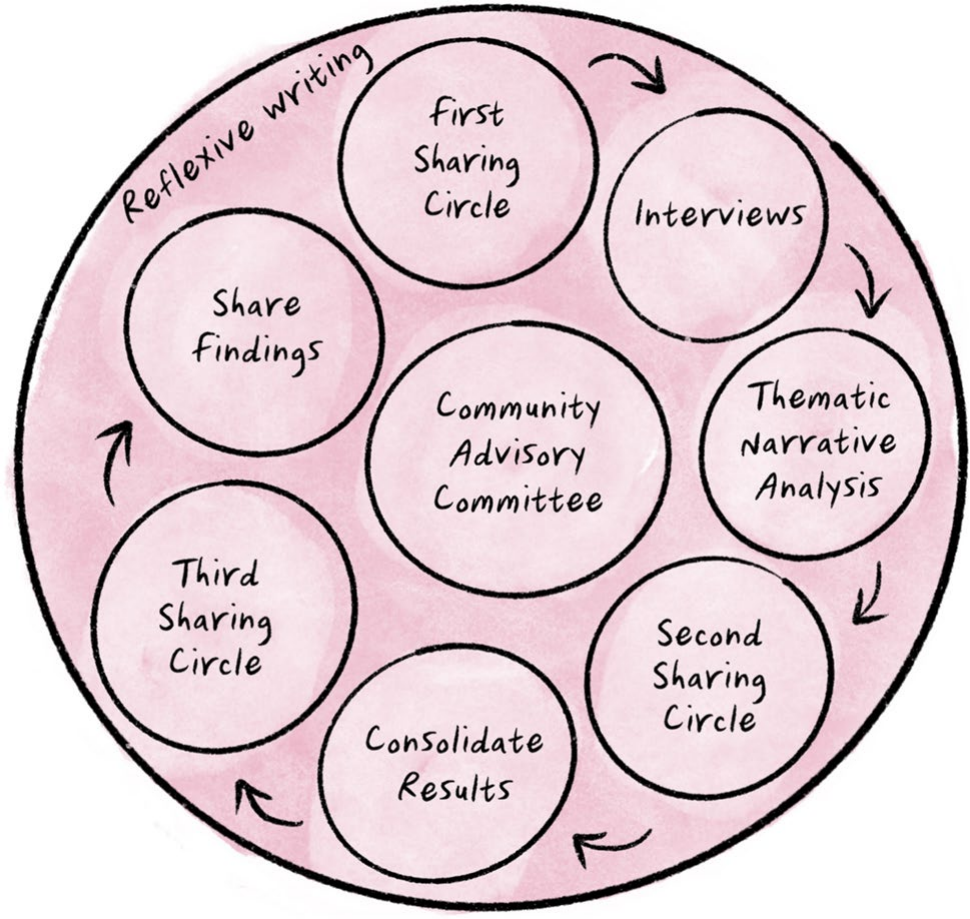
Research design

In collaboration with the CAC, a research design was co-created that included semi-structured interviews with Indigenous Elders, patient advocates, biomedical healthcare providers, policy makers, and hospital administrators, as well as iterative sharing circles with Indigenous Elders (see Fig. 1). The aim of the interviews was to gain a rich diversity of stories, experiences, beliefs, and perspectives about the Indigenous wellness services at the hospital with the goal of improving care for Indigenous patients. As such, we used purposive and snowball sampling to recruit participants who had different relationships to the hospital and distinct cultural identities, community affiliations, genders, and ages (Creswell, 2007; Patton, 1990).

Recruitment and interviews were carried out by a non-Indigenous researcher (SR) who had training and experience conducting ethical and community-engaged research. She had been working at a community-based research institute in Yellowknife for three and a half years before starting the interviews and had collaborated with the former Elders’ Advisory Council at STH. To recruit participants using purposive sampling, SR reached out to individuals who held roles at the hospital, community organizations, and health centres to see whether they were interested in participating in an interview and whether they could share the study information with others. Participants were recruited until saturation was achieved (i.e., no new concepts emerged). Because of the COVID-19 pandemic, all interviews (July–Dec 2020) took place by telephone or videoconference. Most interviews were 1 hour in length, but some ranged from 25 min to 2 hours, depending on participants’ desires and availabilities. The interview guide was co-developed with the CAC and sharing circle Elders. During the interviews, patients and healthcare providers were respectively asked about their experiences receiving and providing care at STH and about their experiences with the Indigenous wellness services.

Altogether, 41 individuals participated in interviews (see Table 1), 19 of whom self-identified as Indigenous. In keeping with our commitment to confidentiality and the advice of the CAC, each participant was given a pseudonym in Dëne Sułıne that aligned with a bird, insect, or animal in the NT. Given concerns about identification of individuals, the demographics reported in Table 1 only include participants’ relationships to STH.

**Table 1:** Interview participants

Number of participants	Role in relation to Stanton Territorial Hospital
13	Elders and patient advocates
16	Healthcare providers at STH
6	Indigenous Wellness Program staff
6	Policy makers and hospital administrators

SR also participated in three sets of sharing circles with four Indigenous Elders in the NT. In early conversations about the study, CAC members indicated that it was important to have Elders from the community with rich experiences of Indigenous healing and hospital care interpret and validate the research findings. Through a series of conversations with the CAC and community Elders, we determined that the CAC would provide oversight and guidance on the research processes (i.e., recruitment, study design and methods, research governance), and four community Elders would be invited to participate in sharing circles to validate the substantive research findings and ensure the appropriate and respectful use of Indigenous knowledge in the results.

Sharing circles are akin to focus groups, a qualitative method where a small group meets to discuss a subject chosen by the researcher; however, they differ from focus groups because of the spiritual significance they hold for many Indigenous Nations and communities (Lavallée, 2017). We called the circles “sharing circles” to reflect the language used by the Elders. The first sharing circle took place prior to the interviews. During the first sharing circle, a draft of the interview questions was shared with the Elders for their reflections, and the questions were revised based on the Elders’ responses. The second sharing circle occurred after we conducted a thematic narrative analysis of the interview data and served to validate and extend the findings, and the third took place once the findings were consolidated and helped inform the methods for knowledge translation (see Fig. 1; all figures in this article © 2021 Rebecca Roher, published with permission). The sharing circles played a key role in the research design because they helped ensure that the findings were relevant to and respectful of Indigenous patients’ experiences.

### Analysis

A thematic narrative analysis of the data from the interviews and first sharing circle was then carried out. The analysis was led by SR and supported by CAC members (including co-authors PA and KF) and team members (JG, TG, SC, and AMP). While narrative analysis tries to examine and preserve the whole story that a participant tells, thematic analysis often pulls apart participants’ responses to identify, analyze, and report patterns of meaning (Kovach, 2010; Martin, 2009). We embraced the tension between these approaches and used both strategies to analyze the data. That is, we drew upon narrative analysis by asking certain questions of the data such as the following: Why is this story being shared? What is the moral of this story? How does the listener’s role influence the telling? Additionally, we used strategies from thematic analysis to organize the data by paying attention to certain concepts and themes that surfaced across participants’ responses.

The analysis included the following steps, which are consistent with general qualitative approaches (Kovach, 2010; Martin, 2009): (1) familiarizing oneself with transcripts from the interviews and first sharing circle; (2) identifying a set of topical codes from individual quotations, stories, and the overall tone and messages from each interview based on key concepts; (3) grouping topical codes into code families; (4) looking for patterns and relationships between the topical and family codes; and (5) once there was a clear draft of the findings, presenting the themes to the Elders in the second sharing circle to validate the findings.

## Results

**Fig. 2:**
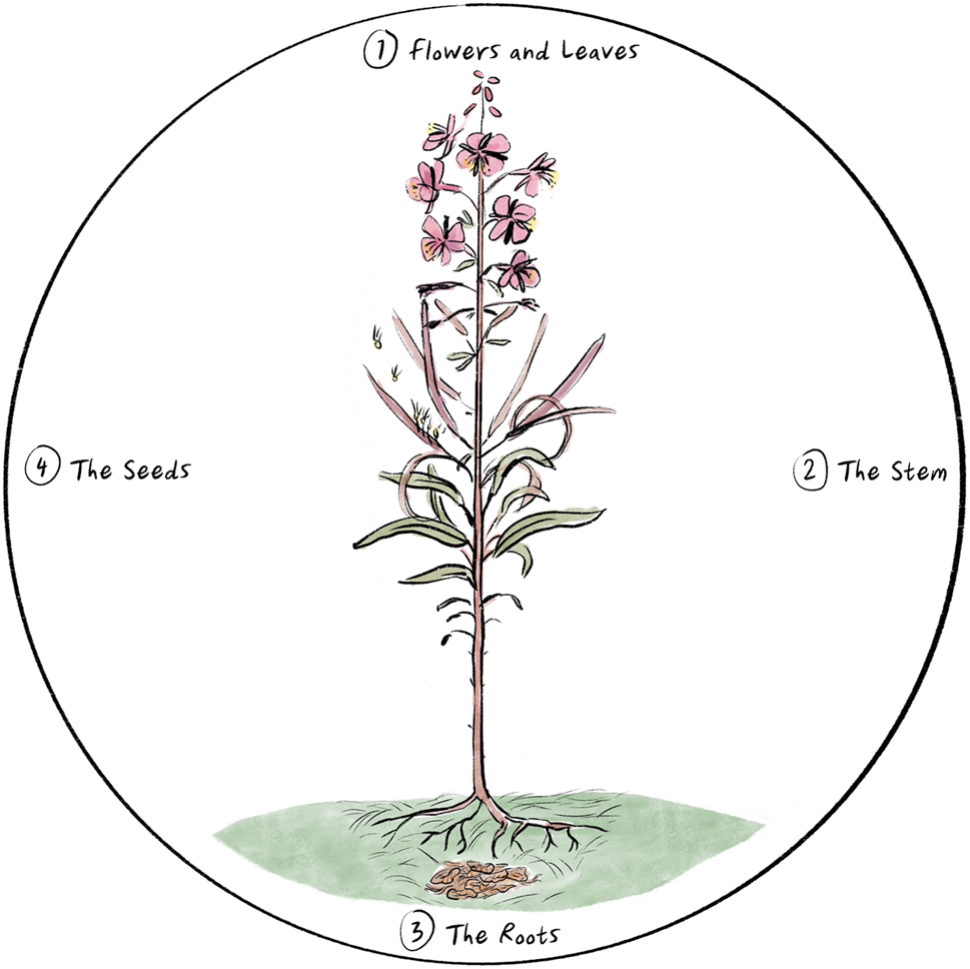
The fireweed model

The image of a fireweed emerged during the analysis as a conceptual model for thinking about the relationships between the study’s themes (see Fig. 2). The fireweed surfaced from participants’ responses. It was initially visualized by SR and was shaped and validated by the Elders in the sharing circles and the CAC members. The fireweed model acts similarly to the iceberg analogy and comparable visual metaphors that have been taken up in Indigenous health research (Absolon, 2011; Reading & Wein, 2009). It provides a visual depiction of the visible and invisible forces that shape Indigenous patients’ experiences with the Indigenous wellness services at STH.

The fireweed model has four elements: (1) the flowers and leaves represent Indigenous patients’ experiences with the Indigenous wellness services at STH; (2) the stem represents organizational structures at the hospital that shape Indigenous patients’ experiences with the wellness services; (3) the roots signify deep-seated forces that underlie organizational structures (i.e., the stem) and inform Indigenous patients’ experiences (i.e., the flowers and leaves); and (4) the seeds represent how participants envisioned Indigenous healing practices successfully working with biomedical hospital care in the future. In this article, we focus on findings that correspond to the flowers and leaves, stem, and roots because these three elements reveal how participants experience the current Indigenous wellness services at STH. A description of the findings that correspond to the fireweed’s seeds can be found in a separate publication (Roher et al., 2023).

### Indigenous patients’ experiences (i.e., the flowers and leaves)

#### The Indigenous wellness services play a key role connecting patients with cultural supports

Elders and patient advocates emphasized that the Indigenous wellness services at STH play an important role connecting patients with cultural supports, such as traditional foods, drumming, and the opportunity to speak Indigenous languages. The Indigenous wellness services’ focus on strength, positivity, culture, and feeling good was viewed as fundamental to healing and feeling safe at the hospital. For example, an Elder shared about a time when the Indigenous Wellness Program staff played drum music for her and her sister at the hospital:

*So that was really good. We watched people drum dancing, and traditional songs, the hand games, oh that was really uplifting!...The tie to our culture is really strong. It’s just enriching. And I know, it gives you a sense of identity and pride in who you are (Ts’i, Elder).*

In a hospital environment, which often focuses on illness and death, the Elder shared that the Indigenous Wellness Program’s cultural supports provided the motivation and healing that she and her sister needed to feel good and feel “alive.” It is not that the cultural supports “healed” her sister by curing her cancer; rather, they brought her healing on a spiritual and emotional level. The connection between Indigenous culture and healing was emphasized by all Indigenous Elders and patient advocates in this study. Participants identified traditional foods, being able to speak in Indigenous languages, and visits with Indigenous Wellness Program staff members as particularly important services because they help patients feel “good” and connected to their culture, which promotes healing.

#### The Indigenous wellness services are insufficient

Participants reported that the way the Indigenous wellness services are currently provided does not meet the needs of many Indigenous patients or meaningfully bring Indigenous healing practices into hospital care. For example, some participants described that the Indigenous Wellness Program staff only work weekdays from 8:30 am to 4:30 pm, which is not adequate because many Indigenous patients require certain services, such as interpretation and navigation, at all hours (Nagídhe, Elder; Yutthę́ ɂejëré, patient advocate). Participants also highlighted that Indigenous patients should be able to eat traditional foods for every meal at the hospital (Sas dełzën and Mułtsagh, Elders; Ɂurádzı, TThełjus, and Ɂı̨yes Ɂeldél, healthcare providers), and they described wanting to see Indigenous counsellors and use Indigenous medicines while at the hospital, but not being able to access them or not being permitted to use them (Ts’ı, Elder; Yutthę́ ɂejëré and Datsą́, patient advocates). Additionally, although healthcare providers often request interpreters, some healthcare providers reported that they or their colleagues did not know about or did not know how to access the Indigenous Wellness Program and its interpretation services (Ts’ı, Elder; Yutthę́ ɂejëré and Datsą́, patient advocates).

### Organizational factors (i.e., stem themes)

#### The lack of Indigenous governance at the hospital

Elders and patient advocates described the lack of Indigenous governance at the hospital and not having an Elders’ Advisory Council as key barriers to meaningfully bringing Indigenous healing practices into hospital care. They relayed that the Indigenous wellness services are not meeting the needs of many Indigenous patients because Indigenous peoples are not able to determine what Indigenous wellness services look like at the hospital. Instead, the wellness services are directed and governed by non-Indigenous peoples and structures who do not understand the realities and values of Indigenous peoples. For many Elders and patient advocates, the lack of Indigenous governance at the hospital helped explain why there was no daily traditional foods program at the hospital, why the Indigenous Wellness Program staff are not working enough hours, and why important aspects of Indigenous healing practices are not included at the hospital, such as Indigenous counsellors and medicines.

*There should be an Aboriginal organization overseeing [the Indigenous Wellness Program] so that [the program] is directed towards the [Indigenous] patients as opposed to whatever the white people think they should be doing (Yutthę́ ɂejëré, patient advocate).*

#### Hospital policies embedded in a Western biomedical perspective

Elders and patient advocates suggested that the hospital is not able to meaningfully bring Indigenous healing practices into hospital care because the Indigenous wellness services and healing practices are expected to conform to the hospital, territorial, and federal policies and standards, which come from a Western biomedical perspective. For example, some participants interpreted the hospital’s policy of *only* allowing smudging in the non-denominational space as an example of how the hospital’s policies expect Indigenous healing practices and ways of knowing to conform and fit into biomedical standards of care.

*There’s no room, there’s no space within that [hospital] system to have our [Indigenous] health system in place. In other words, we’d have to conform. We’d have to juggle this and juggle that to meet the health standard in Canada (Nunıye, Elder).*

#### Senior hospital administration not prioritizing Indigenous wellness services

Healthcare provider participants also offered numerous examples of how the Indigenous Wellness Program was not seen to be a priority for senior hospital administration. Low visibility of the program in the day-to-day functioning of the hospital and not consistently including the Indigenous Wellness Program in healthcare providers’ orientations to the hospital were cited as key examples (Det’áne, Ɂı̨yes Ɂeldél, Deníye, Łue, TThełjus, healthcare providers). Altogether, they culminated in a sense that Indigenous wellness is not prioritized and not valued by senior hospital administration.

### Deep-seated forces (i.e., root themes)

#### Racism and colonialism underlie mainstream healthcare

Some participants saw the lack of Indigenous governance and restrictive policies at the hospital as part of a long history of racism and colonialism in mainstream healthcare delivery in the NT where Indigenous peoples have been left out of decision-making or have been actively silenced. Participants used the recent history of the disbandment of the Elders’ Advisory Council and the continued lack of Indigenous governance at the hospital as clear examples of a colonial relationship where Indigenous peoples are not able to determine their own healthcare needs.

*What was so fascinating about the new hospital and the dynamics around the Elders’ Council is all those [colonial] power dynamics were playing out in full view for everyone to see…We had an opportunity to build a hospital that is actually congruent with where we live and the cultural history, and it was totally missed. In fact, it was damaging (Ɂı̨yes Ɂeldél, healthcare provider).*

#### The dominance of the biomedical model

Participants’ responses illuminated that while Indigenous perspectives see Indigenous culture and wellness services as *essential* to healing and feeling safe at the hospital, the biomedical model sees Indigenous wellness services as *secondary* to the hospital’s primary mission of attending to the physical “health” and “safety” of patients, and sometimes the biomedical model sees Indigenous wellness as an invalid form of healing altogether (Det’anchogh, Sas dełzën, and Nunıye, Elders; Yutthę́ ɂejëré and Datsą́, patient advocates). The different understandings of “healing” and “safety” and the dominance of the biomedical model helped explain why the Indigenous wellness services are expected to conform to the hospital’s Western policies and why the Indigenous wellness services are not a priority for senior hospital administration.

#### Community strengths that promote healing

Elders and patient advocates emphasized strengths in their communities and in the healthcare system, which have helped keep Indigenous peoples healthy and safe at the hospital despite the toxicity of racism and colonialism and the dominance of the biomedical model. Participants highlighted strengths in Elders’ teachings and Indigenous ways of caring for one another; the self-determined approach of the AIWF; and healthcare providers’ openness and willingness to learn from and build relationships with Elders and Indigenous counsellors.

*We are guided by Elders from the past…we have been guided by our Elders and our people. They gave us that strength and knowledge to make sure that when we’re going to do something…[to care] for our people, we’ve got the blessings from our Elders to do it (Sas dełzën, Elder).*

## Discussion

This is the first study to examine hospital-based Indigenous wellness services in the NT. Though the NT is a culturally and contextually distinct region, many of the findings align with existing literature on hospital-based Indigenous wellness services (Maar, 2004; Maar et al., 2009; Maar & Shawande, 2010; Manitowabi & Shawande, 2013; Walker et al., 2010). For example, study participants underlined the importance of having Indigenous governance at the hospital. Indigenous scholars, Elders, and Knowledge Holders have emphasized time and time again that Indigenous peoples need to be involved in healthcare governance and decision-making, particularly if Indigenous ways of knowing are going to be brought into biomedical healthcare settings (Auger et al., 2016; Maar & Shawande, 2010; Royal Commission on Aboriginal Peoples, 1996), and they have demonstrated that Indigenous leadership is vital to providing culturally safe treatment options (Maar & Shawande, 2010; Manitowabi & Shawande, 2013; Walker et al., 2010). Our study is the first to our knowledge that examined Indigenous wellness services in a mainstream hospital environment in Canada where there was no Indigenous governance. Nevertheless, our results support previous study findings by revealing the negative impacts of insufficient or no Indigenous governance. Similarly, the findings support work carried out by Indigenous and non-Indigenous scholars about racism and oppression in healthcare systems in Canada (Allan & Smylie, 2015; Reading & Wein, 2009; Turpel-Lafond, 2020), demonstrating that to improve care for Indigenous patients, structural and systemic changes are needed that address racist practices and policies and move towards greater Indigenous self-determination.

Our research also offers new insights that deepen the existing literature on Indigenous wellness services in mainstream Canadian hospitals. For example, some of the key contributions of this project are the conceptual and causal relationships between the flowers and leaves, stem, and roots that were revealed through the study and represented through the fireweed model (see Table 2 for examples of these relationships). Participants’ responses demonstrated that deep-seated ways of thinking and doing (i.e., the roots) shape the development of organizational structures (i.e., the stem) and underlie Indigenous patients’ experiences (i.e., the flowers and leaves). While previous studies examining hospital-based Indigenous wellness services make mention of some of the themes surfaced in this study, such as racism and colonialism (Achan et al., 2021), biomedical dominance (Manitowabi & Shawande, 2013), and the importance of Indigenous governance (Drost, 2019; Maar & Shawande, 2010; Manitowabi & Shawande, 2013; Walker et al., 2010), they do not explicitly describe the relationships between them. In this way, the fireweed model acts as both a tool to communicate findings and a conceptual model to deepen understandings of hospital-based Indigenous wellness services.

**Table 2:**
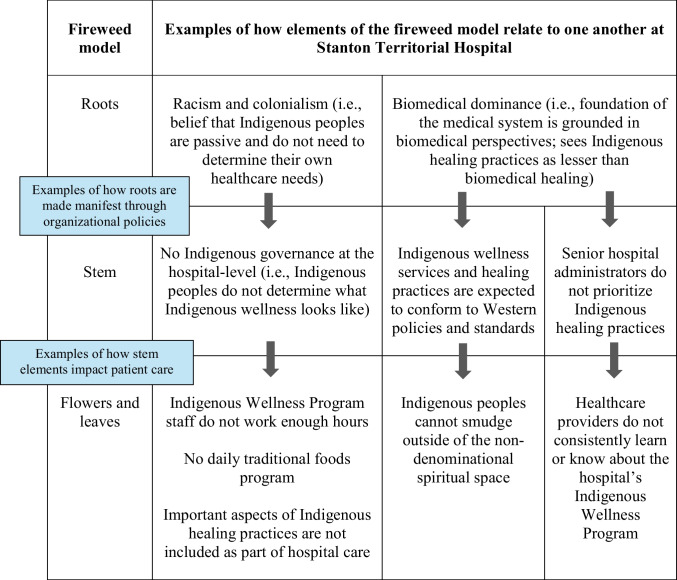
Conceptual and causal relationships that were revealed through this study and represented through the fireweed model

The conceptual and causal relationships in the fireweed model provide important insights for future public health research and practice. Healthcare providers and public health practitioners are at risk of reproducing colonial ideologies if they do not develop the skills they need to critically examine and deconstruct biomedical dominance, racism, and colonialism underlying healthcare practices (Beavis et al., 2015). The fireweed model can help mitigate this concern by providing an easily understandable visual image, which illustrates the complex relationships between different deeply rooted forces, organizational structures, and Indigenous patients’ experiences.

The fireweed model also points to sustainable and long-term directions to improve care for Indigenous patients at STH. For example, the relationships demonstrate that toxic roots underlie hospital care delivery and that it is critical to unearth and address the unhealthy roots (i.e., racism, colonialism, and biomedical dominance) to advance patient care. Similarly, the findings reveal how “stem” elements (i.e., the hospital’s policies and processes) can play a mediating role at the hospital between deep-seated forces and ideologies (i.e., the roots) and Indigenous patients’ experiences (i.e., the flowers and leaves). The stem elements can act as manifestations of the roots. They can be one way in which the roots are enacted and enforced in a hospital’s organizational structures. As a result, to improve care for Indigenous patients, the findings illustrate that it is critical not only to address toxic roots, but also to parse out the ways that toxic roots may have become solidified and reproduced through organizational structures, policies, and processes.

Additionally, the findings suggest that to improve care for Indigenous patients, STH must focus attention and resources towards supporting and building healthy strength-based roots, such as Elders’ teachings, Indigenous self-determination, and fostering relationships between Elders and biomedical healthcare providers. In this way, instead of becoming stuck in one way of being and/or only paying attention to the negative and toxic root system, the fireweed model allows for change, growth, and flexibility, and provides avenues for possible stronger futures to emerge.

### Limitations and future research

Since recruitment and data collection for the project took place within the COVID-19 pandemic, we did not solicit the participation of certain under-represented populations, such as Indigenous peoples in the NT who did not speak English and individuals who did not have access to videoconference or telephone. Similarly, the research did not include the experiences of minors (under 18 years old) or residents of Nunavut who access care at STH. Future research should include the knowledge and experiences of these populations.

Additionally, the study findings illuminate the need for more published research on hospital-based Indigenous wellness services. Though scholarship on hospital-based Indigenous wellness services in Canada is growing, it is still very limited. Future research could examine whether the conceptual and causal relationships, which were illuminated in this study and reflected in the fireweed model, are relevant to other hospital settings or other institutional environments where Indigenous and non-Indigenous ways of knowing are commonly brought together (i.e., education, justice). Researchers might ask: What are the different factors (organizational, structural, systemic, ideological) that shape Indigenous peoples’ experiences in these settings? To what extent might these factors relate to one another? And, what images (if any) might help to convey these relationships? These responses could then be compared across study regions and contexts and provide insights for further learning.

## Conclusion

The research findings extend the literature on hospital-based Indigenous wellness services by demonstrating the connections between underlying belief systems, organizational structures, and patient experiences, and they support calls made by the TRC and UNDRIP for Indigenous knowledges and healing practices to be *valued* and for Indigenous peoples to have control over their own healthcare services and healing practices (Truth & Reconciliation Commission of Canada, 2015; United Nations General Assembly, 2007). When examined altogether, the findings highlight that Indigenous peoples and communities need to be the ones to determine and define whether Indigenous wellness services are offered alongside hospital care and how those services are provided; this is critical to Indigenous self-determination and to improving care for Indigenous patients.

## Contributions to knowledge

What does this study add to existing knowledge?

- First study to examine hospital-based Indigenous wellness services in the Northwest Territories.
- Extends understandings of hospital-based Indigenous wellness services by surfacing relationships between deeply rooted forces, organizational structures, and Indigenous patients’ experiences.
- Reveals the negative impact of insufficient Indigenous governance at a hospital.

What are the key implications for public health interventions, practice, or policy?

- Provides a conceptual model for thinking about hospital-based Indigenous wellness services that points to sustainable and long-term directions to improve care for Indigenous peoples.
- Demonstrates the ways that colonialism, racism, and self-determination intersect with healthcare programming and structures and act as key determinants of health for Indigenous peoples and communities.
- Underscores that Indigenous peoples and communities need to be the ones to determine and define whether and how Indigenous knowledges and healing practices are brought into health services, interventions, and policies.
